# Identification of metabolic reprogramming-related key genes in hepatocellular carcinoma after transcatheter arterial chemoembolization treatment

**DOI:** 10.1007/s12672-025-02606-z

**Published:** 2025-05-22

**Authors:** Tongfei Li, Shujuan Liu, Shengjun Wang, Shan Sun, Feng Ji, Mingliang Li, Yong Zhang

**Affiliations:** 1 Department of Interventional Radiology, The Second Affiliated Hospital of Shandong First Medical University, Tai’an, 271000 China; 2 Department of Oncology, Jinan Seventh People’s Hospital, Jinan, 250132 China; 3 Department of Oncology, Shanxian Dongda Hospital, Heze, 274399 China; 4 Automation Department, Jigang Group International Engineering Technology Co., Ltd, Jinan, 250098 China; 5 School of Preventive Medicine, Shandong First Medical University, No. 6699 Qingdao Road, Jinan, 250117 Shandong People’s Republic of China

**Keywords:** Hepatocellular carcinoma, Transcatheter arterial chemoembolization, Treatment sensitivity, Metabolic reprogramming, Biomarkers

## Abstract

**Background:**

Metabolic reprogramming plays an important role in therapeutic efficacy of hepatocellular carcinoma (HCC). However, the metabolic reprogramming-related key genes associated with transcatheter arterial chemoembolization (TACE) treatment sensitivity in HCC remain further investigation.

**Methods:**

We analyzed data from public databases, The Cancer Genome Atlas and Gene Expression Omnibus, as well as metabolism-related genes (MRGs), to identify key genes associated with TACE treatment sensitivity. Further analysis was conducted on the relationship between key genes and immune cell infiltration, HCC-related genes, regulatory network construction, nomogram construction, and drug sensitivity analysis. Finally, the expression of key genes was validated based on databases and in *vitro* RT-qPCR.

**Results:**

Four key genes (CDC20, LPCAT1, PON1, and SPP1) associated with TACE treatment sensitivity were identified. Increased CDC20, LPCAT1, and SPP1 and reduced PON1 were found in tumor tissues than normal tissues, as well as in advanced patients than early-stage patients. Lower expression of CDC20, LPCAT1, and SPP1, and higher expression of PON1 were detected in responsive patients than non-responsive patients. Patients with high expression of CDC20, LPCAT1, and SPP1, and low expression of PON1 had poor prognosis. They were also correlated with tumor immune microenvironment and sensitivity to multiple chemotherapy drugs. The expressions of key genes at the gene and protein levels were validated.

**Conclusions:**

Our study provided systematic insights into identification of biomarkers for TACE treatment sensitivity in HCC.

**Supplementary Information:**

The online version contains supplementary material available at 10.1007/s12672-025-02606-z.

## Introduction

Hepatocellular carcinoma (HCC) is the malignancy with the highest incidence among primary liver cancers worldwide [1]. Due to its unclear early symptoms and generally poor prognosis, it faces significant challenges in treatment [2]. At present, the treatment strategy for HCC has developed into a multidisciplinary comprehensive treatment centered on surgical procedures, including ablation therapy, interventional therapy, targeted therapy, and immunotherapy [3]. Among them, transcatheter arterial chemoembolization (TACE) is widely used as an interventional therapy for advanced HCC patients, controlling tumor growth by blocking the tumor blood supply [4]. However, the reduced sensitivity of TACE treatment brings certain limitations and challenges, especially after multiple TACE treatments. It was recommended to abandon further treatments for patients who remained non-responsive after three TACE treatments, as the subsequent effectiveness rate would be less than 10% [5]. Therefore, improving the TACE treatment sensitivity has become an urgent problem to be solved.

Metabolic reprogramming, as one of the key mechanisms for HCC treatment resistance, is an adaptive strategy for tumor cells that adjusts their energy requirements and metabolic pathways to make them flexibly respond to changes in growth and survival environment [6, 7]. Glutamine metabolism was promoted by aspartate aminotransferase 2 (GOT2) silencing, making HCC sensitive to glutaminase inhibitors [8]. Glycolysis energy metabolism was promoted by BCL2 interacting protein3 (BNIP3) -mediated mitophagy, thereby enhancing the competitive growth of lenvatinib-resistant HCC cells [9]. Lipid metabolism reprogramming of HCC cells promoted resistance for sorafenib-induced ferroptosis in the presence of reduced lncRNA HNF4A-AS1 [10]. It has been reported that TACE treatment sensitivity in HCC is associated with enhanced glycolytic activity due to down-regulation of fructose-1, 6-bisphosphate aldolase B (ALDOB) [11]. Another study found that pharmacological inhibition of lactate dehydrogenase A (LDHA) in the glycolytic pathway can enhance the antitumor efficacy of TACE [12]. Although there are currently clues revealing the regulatory mechanism of metabolic reprogramming involved in TACE treatment sensitivity in HCC, it is still difficult to effectively identify patients with poor TACE treatment response and achieve targeted treatment. Therefore, there is an urgent need to develop a series of biomarkers and elucidate their roles in the TACE treatment response of HCC to help achieve clinical precision therapy.

In this study, we utilized public databases, The Cancer Genome Atlas (TCGA) and Gene Expression Omnibus (GEO), as well as metabolism-related genes (MRGs), to identify key genes associated with TACE treatment sensitivity. Subsequently, we systematically analyzed their relationship with tumor characteristics, the disease progression and survival prognosis of patients, and chemotherapy drug sensitivity. The transcription factor (TF) regulatory network and competitive endogenous RNA (ceRNA) regulatory network were constructed, and the expression of key genes at gene and protein levels was verified. This study will provide an effective theoretical foundation for TACE treatment in HCC.

## Materials and methods

### Data collection and processing

All gene expression data were obtained from public databases and processed using R (version 3.5.3) software (https://www.r-project.org/). The RNA-seq data (TCGA-LIHC.htseq_fpkm.tsv) of The Cancer Genome Atlas-Hepatocellular carcinoma (TCGA-LIHC), matched survival information (TCGA-LIHC.survival.tsv), and clinicopathological characteristics data (TCGA-LIHC.GDC_phenotype.tsv) were extracted from the Xena database (https://xena.ucsc.edu/) of the University of California, Santa Cruz (UCSC). After excluding patients without survival information, survival information and expression data of tumor tissue samples were integrated to obtain 365 TCGA-LIHC samples for prognostic analysis. The RNA annotations file in GENCODE (https://www.gencodegenes.org/) was used to obtain the mRNA expression matrix.

The GSE104580 (https://www.ncbi.nlm.nih.gov/geo/query/acc.cgi?acc = GSE104580) dataset was downloaded from the Gene Expression Omnibus (GEO) database, which included 66 TACE non-responder samples and 81 TACE responder samples. Probes were annotated to genes using GPL570 platform files. For genes corresponding to multiple probes, the mean expression value was taken as the gene expression value.

The metabolism related gene (MRG) list was obtained from GeneCards (https://www.genecards.org/) by using "Metabolism" as the search term [13].

### Identification and functional enrichment analysis of differentially expressed genes (DEGs)

The R package “limma” (version 3.36.5) [14] was applied to identify the DEGs between tumor and normal tissues, as well as between the TACE response and non-response groups. The screening criteria for DEGs were |Log_2_Foldchange|≥ 1 and adjusted *P* value < 0.05 [15]. The DAVID database (https://david.ncifcrf.gov/) [16, 17] was used for functional enrichment analysis, including Gene Ontology (GO) and Kyoto Encyclopedia of Genes and Genomes (KEGG) enrichment analysis (adjusted *P* value < 0.05) [18].

### PPI network analysis of DEGs

To uncover the interactions among DEGs, the protein–protein interaction (PPI) network was constructed using the STRING database (https://cn.string-db.org/) with an interaction score setting to 0.4. The PPI network was visualized using Cytoscape (version 3.8.2).

### Identification of key genes

The DEGs obtained from the TCGA-LIHC dataset and GSE104580 dataset were intersected with MRGs to obtain the shared genes. Univariate Cox regression analysis was performed for the shared genes, and the prognostic-related genes were screened with *P* < 0.05 [19]. R package “randomForestSRC” (version 3.3.3) was used for the random survival forest analysis to rank the importance of prognostic-related DEGs [20]. And genes with relative importance > 0.015 were selected for subsequent analysis, including its expression between tumor and normal tissues, TACE response and non-response groups, different stages of disease progression, as well as survival analysis. Statistical analysis was conducted using Wilcoxon test for comparison.

### Immune infiltration analysis

The CIBERSORT algorithm, an innovative deconvolution method based on gene expression, was used to determine differences in immune cell infiltration levels between tumor and normal samples. The method was able to quantify cell composition of complex tissue sample from gene expression profiles, and then to parse the relative proportions of 22 human immune cell types from admixed expression data [21]. The results were visualized in box plots. Additionally, we investigated the correlation between key genes and differentially infiltrated immune cells by Pearson correlation analysis.

### Correlation analysis of key genes and immune related-genes

The immunogene set, including immunoinhibitor, immunostimulator, chemokine, major histocompatibility complex (MHC), and MHC receptor, was downloaded from the TISIDB database (http://cis.hku.hk/TISIDB/index.php). The correlation between key genes and immune genes was explored by Pearson correlation analysis.

### Correlation analysis of key genes and disease-related genes

The HCC-related genes were obtained from the GeneCards database (https://www.genecards.org/) using “Liver hepatocellular carcinoma” as the keyword. The correlation between key genes and HCC-related genes was explored by Pearson correlation analysis.

### Nomogram construction

The R package "rms" (version 6.2–0) [22] was used to construct the nomogram model according to the expression levels of key genes and the clinical characteristics of patients, including age, gender, tumor stage, grade, and TNM stage, in the TCGA-LIHC dataset. The score values corresponding to each predictive indicator can be obtained according to the nomogram, and their sum was recorded as the total score, which corresponds to the total survival probability of 1, 3, and 5 years. The consistency between the expected probability and the actual outcome was assessed by the calibration curve. The R package “ggDCA” (version 1.2) [23] was used for decision curve analysis (DCA) to evaluate the clinical utility of the nomogram model.

### Construction of TF regulatory network

The R package “RcisTarget” (version 1.16.1) [24] was used to predict TFs based on motifs. These motifs could be annotated based on annotated source data files or gene sequences that are similar to annotated motifs. The area under the curve (AUC) of each pair of motif-motif was calculated according to the gene-set recovery curves ranked by motif. Then, the normalized enrichment score (NES) for each motif was calculated based on the AUC distribution for each motif in the gene set. The NES of motif depends on the total number of motifs in the database. hg19-tss-centered-10 kb-7species.mc9nr.feather was used as the motif ranking file.

### Construction of lncRNA-miRNA-mRNA (ceRNA) regulatory network

HCC-related microRNA (miRNA) were obtained from human microRNA disease database (HMDD, http://www.cuilab.cn/hmdd). The miRNAs interacting with key genes were predicted using the miRWalk database (http://mirwalk.umm.uni-heidelberg.de/). Subsequently, the HCC-related miRNAs and predicted-miRNAs from the miRWalk database were intersected to obtain miRNA-mRNA interaction pairs. LncRNA was predicted based on known miRNAs using DIANA-LncBase Predicted v.2 database (https://dianalab.e-ce.uth.gr/html/diana/web/index.php?r=lncbasev2), and those with scores > 0.9, the tissue being liver, and the category being cancer or malignancy were retained. Based on lncRNA-miRNA pairs and miRNA-mRNA pairs, the ceRNA network was visualized by Cytoscape.

### Drug sensitivity analysis

Drug sensitivity information was obtained from the GDSC1 dataset (https://www.cancerrxgene.org/downloads/bulk_download) derived from the Cancer Drug Sensitivity Genomics (GDSC) database [25]. The half-maximal inhibitory concentration (IC_50_) values for drug therapy were calculated using the R package “oncoPredict” [26]. The correlation between key genes and IC_50_ values of drugs was explored by Pearson correlation analysis.

### Verification of key genes

To verify the expression levels of key genes, the GSE25097 (https://www.ncbi.nlm.nih.gov/geo/query/acc.cgi?acc = GSE25097) dataset was downloaded from the GEO database, which included 243 normal and 268 tumor tissue samples. Immunohistochemical (IHC) data were downloaded from the Human Protein Atlas (HPA, http://www.proteinatlas.org) to further validate the protein expression levels of key genes between tumor and normal tissues.

In addition, real-time quantitative polymerase chain reaction (RT-qPCR) was used to detect the expression levels of key genes in blood samples from 15 individuals, including 5 controls, 4 TACE non-responsive HCC patients, and 6 TACE-responsive HCC patients. The clinical information of the subjects was shown in Table S1, and the clinical-pathological characteristics of the TACE treatment response/non-response groups were matched. The study was approved by the Ethics Committee of the Second Affiliated Hospital of Shandong First Medical University (2024-H-059), and all participants provided written informed consent. The total RNA was extracted using the HiPure Liquid RNA Mini Kit (Cat. No.: R4163-02, Magen, Shanghai, China). Reverse transcription was carried out using FastQuant cDNA First Strand Synthesis Kit (Cat. No.: KR106, TIANGEN, Beijing, China) to synthesize cDNA. RT-qPCR was performed using Gene-9660 Fluorescence Quantitative PCR Analyzer (BIOER, Hangzhou, China). The mRNA expression levels were quantified using the 2^−ΔΔCT^ method and analyzed by one-way ANOVA in GraphPad Prism software (version 8.0.1). The primer sequences were shown in Table S2.

## Results

### Identification and functional enrichment analysis of DEGs

**Fig. 1:**
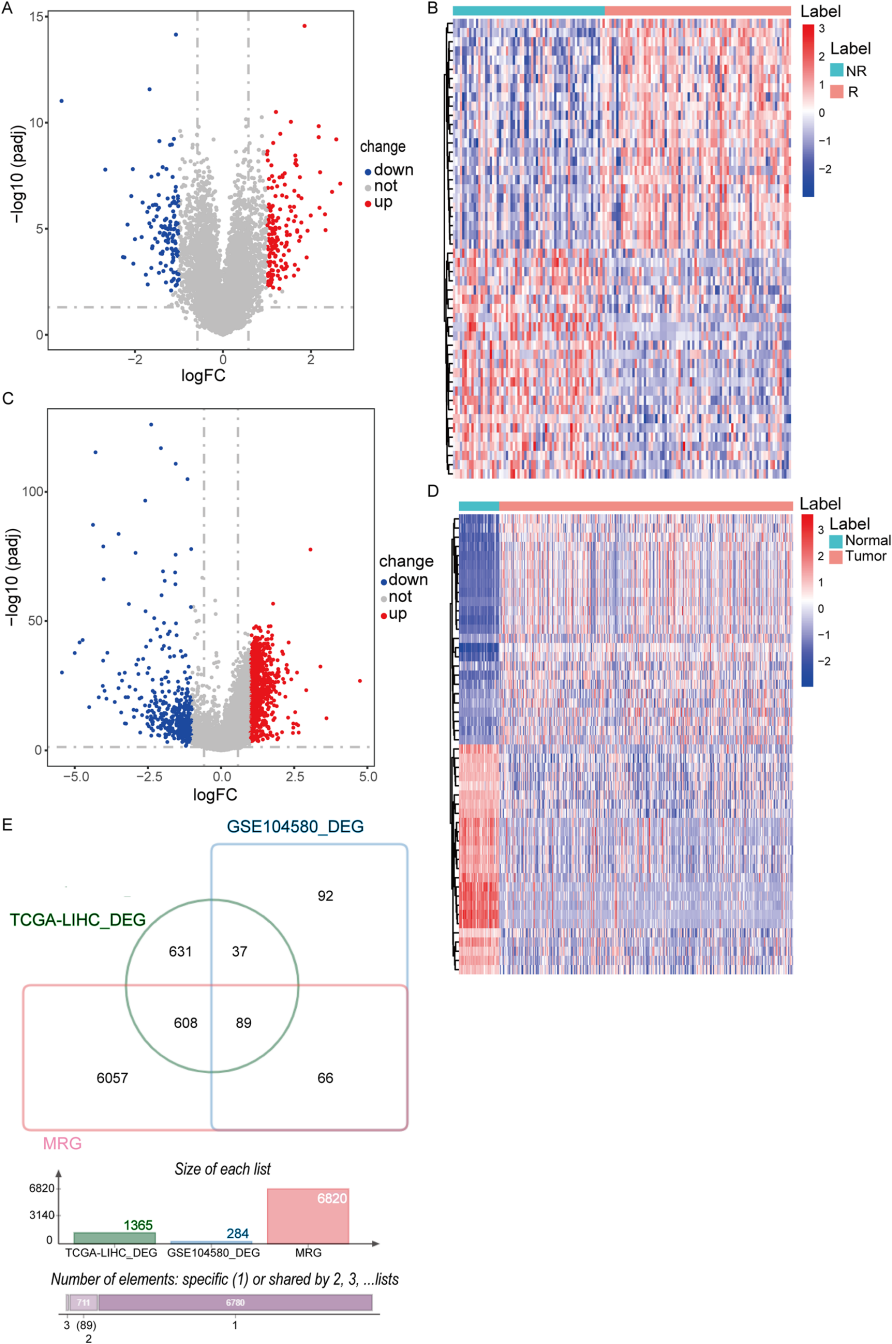
Identification of DEGs. **A** Volcano plot displays the DEGs between TACE responders and non-responders. Red indicates upregulation, blue indicates downregulation. **B** Heatmap shows the expression profiles of top 50 DEGs between TACE responders and non-responders. Red indicates upregulation, blue indicates downregulation. NR, non-responders; R, responders. **C** Volcano plot displays DEGs between tumor and normal tissues. **D** Heatmap shows the expression profile of the top 50 DEGs between tumor and normal tissues. **E** Venn diagram shows the intersection of MRGs with DEGs from the GSE104580 dataset and TCGA-LIHC dataset

To reveal the gene characteristics associated with TACE treatment sensitivity, we performed differential expression analysis on the GSE104580 dataset and identified a total of 284 DEGs between the two groups. Compared to TACE non-responders, 160 genes were up-regulated in TACE responders, while 124 genes were down-regulated (Fig. 1A-B). GO enrichment analysis showed that these DEGs mainly participated in substance metabolism by affecting enzyme activity and function in extracellular and intracellular (Figure S1A). DEGs were mainly enriched in metabolism-related pathways in KEGG analysis (Figure S1B). And PPI network analysis shows that there are complex interactions between most DEGs (Figure S1C).

Subsequently, 1365 HCC-related DEGs were identified from the TCGA-LIHC dataset, of which 975 were up-regulated and 390 were down-regulated (Fig. 1C-D). Then, the DEGs screened out from the TCGA-LIHC and GSE104580 datasets were overlapped with MRGs to obtain 89 shared genes (Fig. 1E).

### Identification of four key genes

**Fig. 2:**
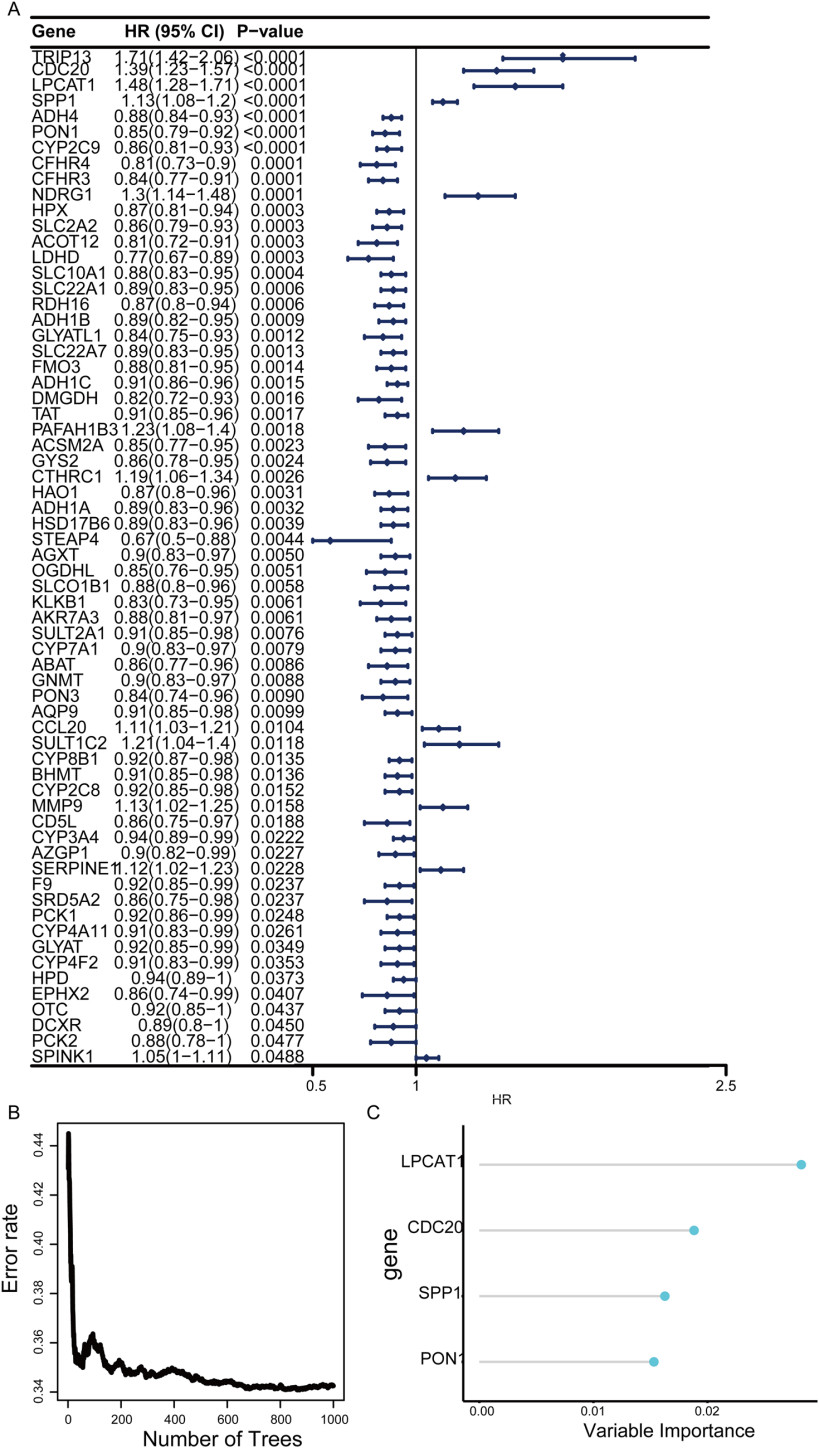
Identification of key genes. **A** Forest plot displays 69 genes associated with prognosis by univariate Cox analysis. HR, Hazard Ratio; CI, Confidence Interval. **B** Random survival forest analysis of 65 genes. **C** Importance lollipop chart displays the importance of the four key genes. The horizontal axis represents the importance score of the gene, the higher the score, the more important

**Fig. 3:**
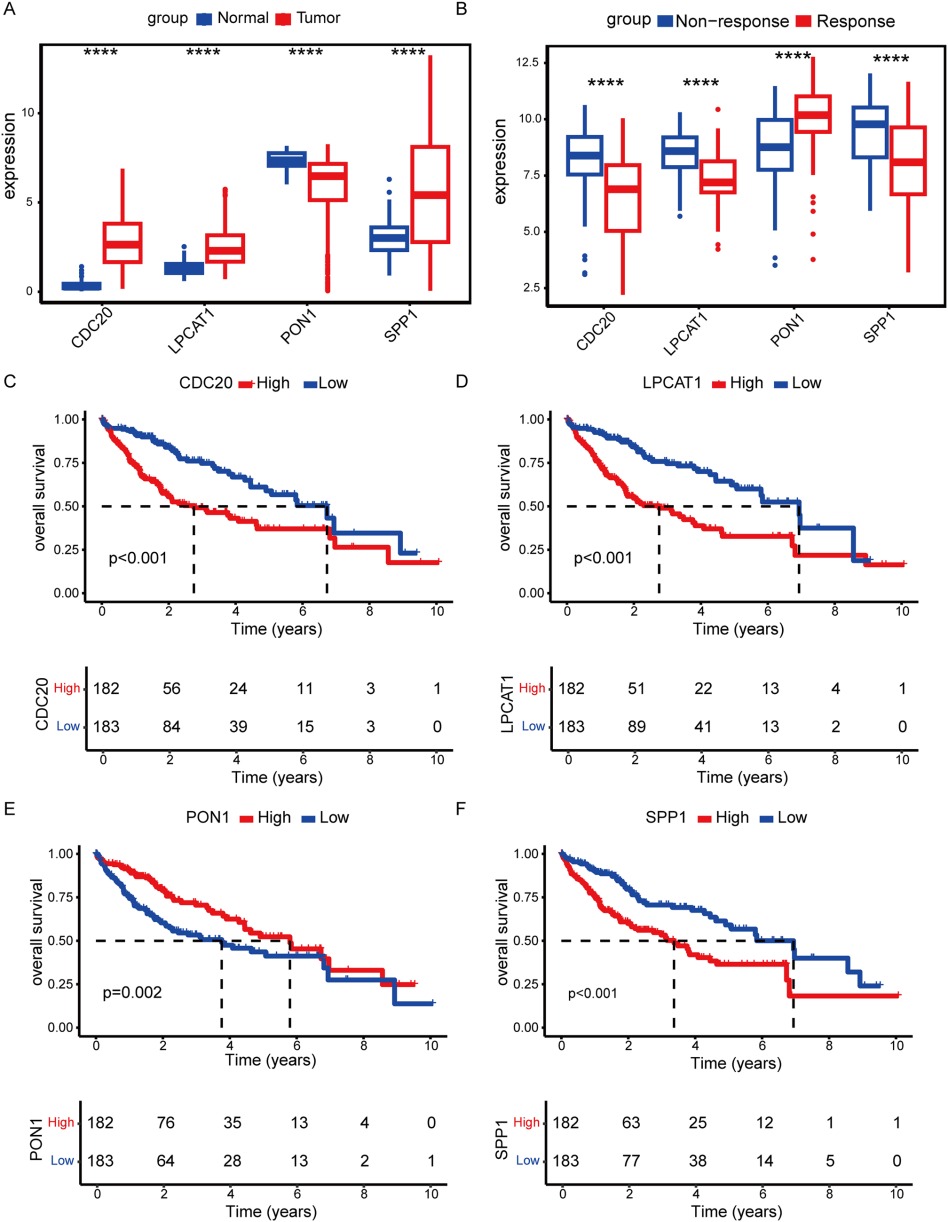
Characteristics of key genes. **A**, **B** Box plot shows the expression levels of four key genes between tumor and normal tissues (**A**), as well as in the TACE treatment response and non-response groups (**B**). ****P < 0.0001. **C**-**F** Kaplan Meier curve analysis for CDC20 (**C**), LPCAT1 (**D**), PON1 (**E**), and SPP1 (**F**)

**Fig. 4:**
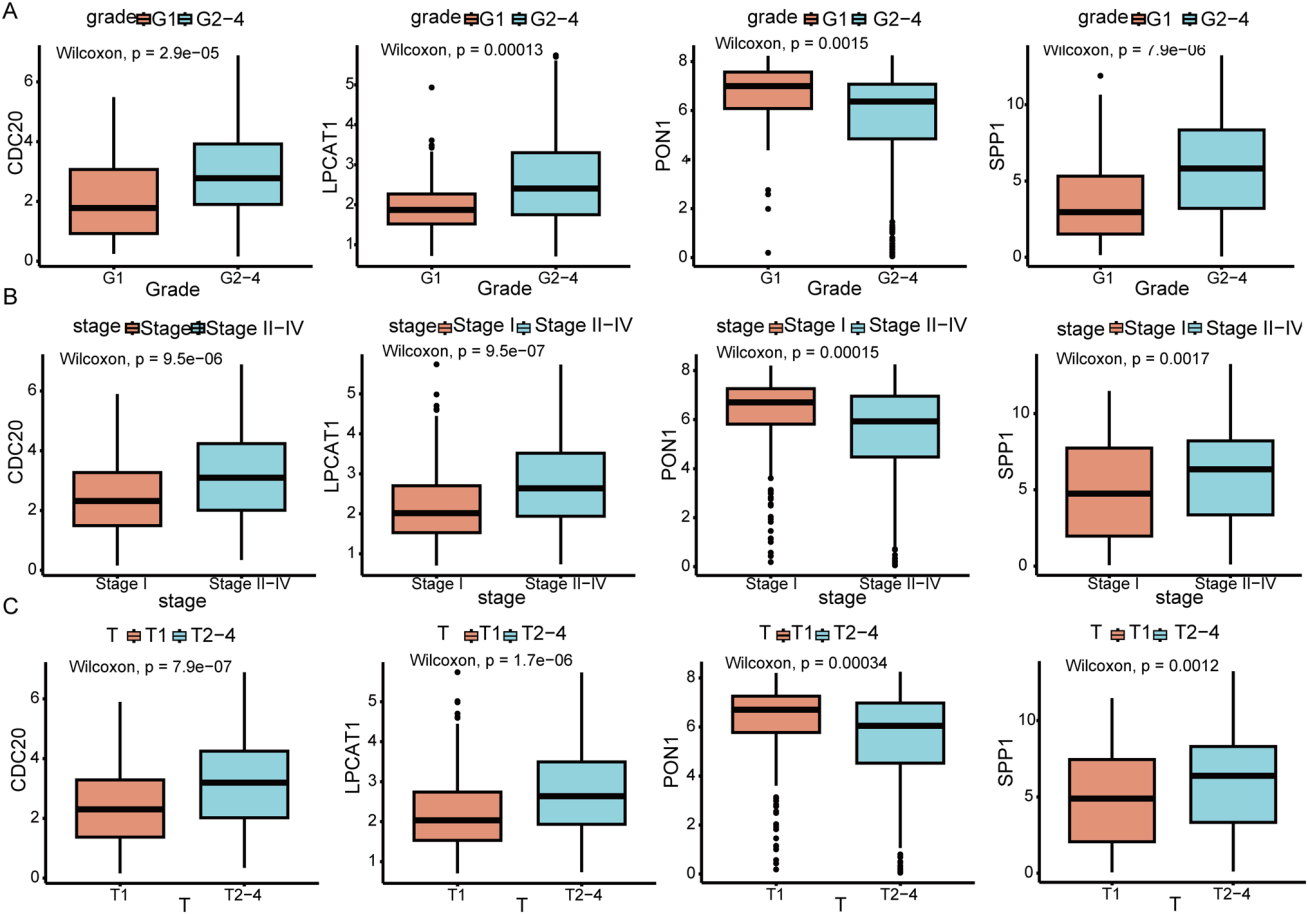
The expression levels of key genes in different stages of tumor grade (**A**), tumor stage (**B**) and T stage (**C**)

In total, 65 prognostic-related genes from 89 shared genes were screened using univariate Cox analysis (*P* < 0.05) (Fig. 2A). Then, four key genes (CDC20, LPCAT1, PON1, SPP1) were ultimately identified through further random survival forest analysis (Fig. 2B-C). The results of TCGA-LIHC dataset analysis showed that compared with normal tissues, CDC20, LPCAT1, and SPP1 were significantly up-regulated and PON1 was significantly down-regulated in tumor tissues (Fig. 3A). Compared with non-responsive HCC patients, CDC20, LPCAT1, and SPP1 expression were significantly lower in responsive HCC patients, while PON1 expression was significantly higher (Fig. 3B). Kaplan Meier survival analysis showed that patients with high expression of CDC20, LPCAT1, and SPP1, and low expression of PON1 had poor prognosis (Fig. 3C-F). Moreover, CDC20, LPCAT1, and SPP1 expression were higher in advanced patients, while PON1 expression was lower (Fig. 4).

### Correlation analysis of key genes with immune microenvironment and immune-related genes

**Fig. 5:**
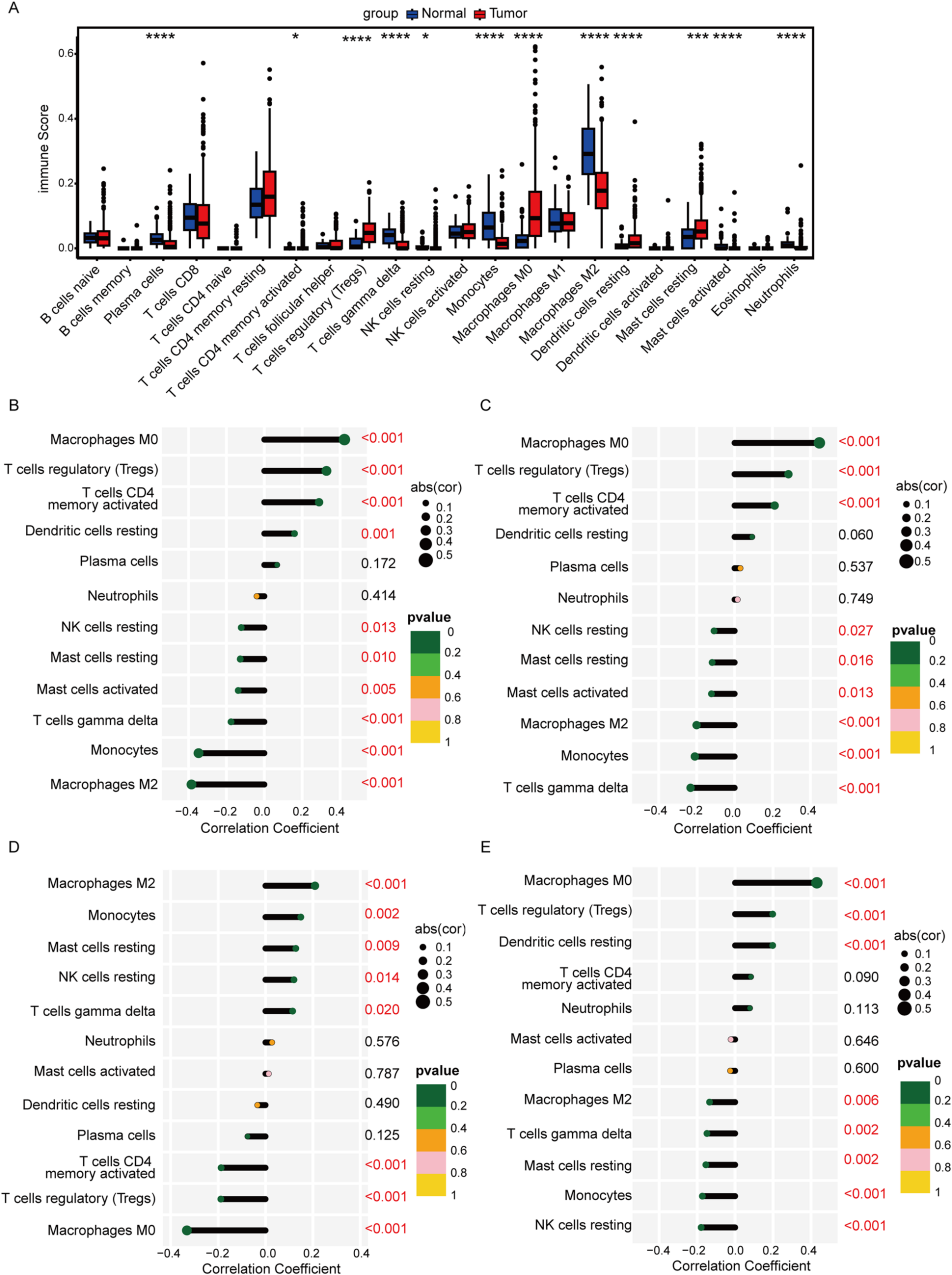
Immune microenvironment analysis. **A** Box plot showing the difference in immune cell infiltration levels between tumor and normal tissues. *P < 0.05, ***P < 0.001, ****P < 0.0001. **B**-**E** Stick plot displays the correlation between CDC20 (**B**), LPCAT1 (**C**), PON1 (**D**), and SPP1 (**E**) and differential immune cells. The horizontal axis represents the value of the correlation coefficient, where > 0 indicates a positive correlation and < 0 indicates a negative correlation. The vertical axis represents the type of immune cells. The circle size reflects the absolute value of the correlation coefficient, and the circle color represents the *P* value of the correlation

The results of immune cell infiltration analysis showed that there were significant differences in the infiltration levels of 12 types of immune cell between tumor and normal tissues (Fig. 5A). Among them, the infiltration levels of "T cells regulation (Tregs)", " Macrophages M0", and "Dendritic cells restoration" were higher, while the infiltration levels of "NK cells restoration", "Monocytes", "Macrophages M2", and "Neutrophils" were lower in tumor tissues. Further correlation analysis revealed that four key genes were significantly associated with multiple immune cells (Fig. 5B-E). CDC20, LPCAT1, and SPP1 expression levels were significantly positively correlated with the infiltration levels of "Macrosphages M0" and "T cell regulators (Tregs)", and significantly negatively correlated with the "Macrosphages M2", "Monocytes", and "NK cell resting". PON1 expression level was significantly positively correlated with the infiltration levels of "Macrosphages M2", "Monocytes", and "NK cells resting", and significantly negatively correlated with the "Macrosphages M0", "T cell regulators (Tregs)", and "T cell CD4 memory activated".

In addition, four key genes were highly correlated with immune-related genes (Figure S2). For example, CDC20, LPCAT1, and SPP1 were significantly positively correlated with the majority of chemokines, immunosuppressants, immune activators, MHC, and MHC receptors, while PON1 was significantly negatively correlated.

### Correlation analysis of key genes with HCC-related genes

**Fig. 6:**
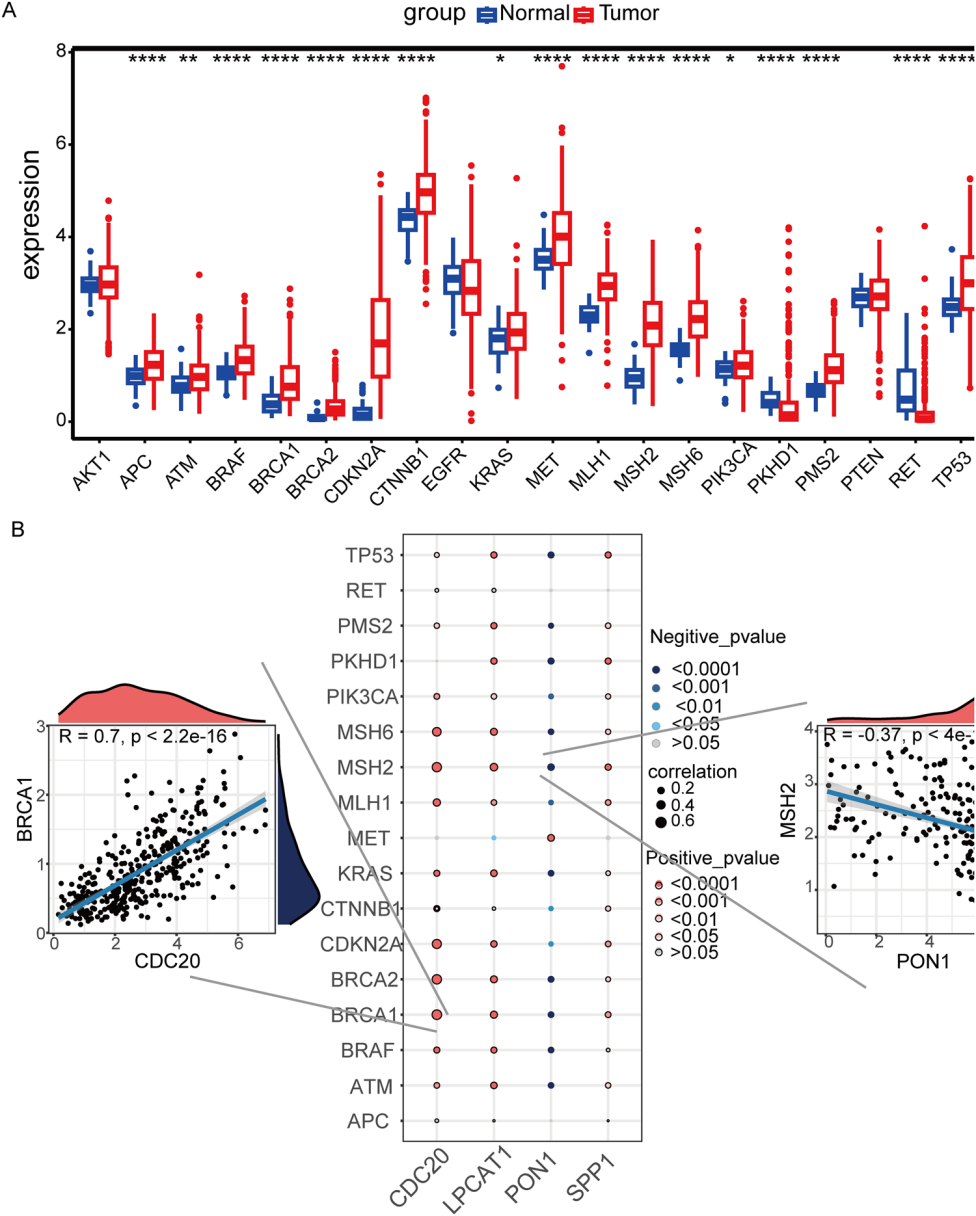
Correlation analysis between key genes and HCC-related genes. **A** Box plot shows expression levels of 17 HCC-related genes between normal and tumor tissues. *P < 0.05, **P < 0.01, ****P < 0.0001. **B** Bubble chart displays the association analysis between the 4 key genes expression levels and HCC-related genes. The size of bubbles indicates the strength of correlation, blue bubbles from deep to light indicate a gradual weakening of negative correlation, and red bubbles from deep to light indicate a gradual weakening of positive correlation

HCC-related genes were obtained from the GeneCards database, and the top 20 genes (relevance score > 105) were retained for subsequent analysis. Among them, 17 genes (APC, ATM, BRAF, BRCA1, BRCA2, CDKN2A, CTNNB1, KRAS, MET, MLH1, MSH2, MSH6, PIK3CA, PKHD1, PMS2, RET, TP53) showed a significant difference in expression between tumor and normal tissues (Fig. 6A). Correlation analysis indicated that the expression of CDC20, LPCAT1, and SPP1 was significantly positively correlated with the multiple HCC-related genes, while the expression of PON1 was significantly negatively correlated (Fig. 6B). The positive correlation between the expression of CDC20 and BRCA1 was the strongest (R = 0.7, *P* < 2.2E-16), while the negative correlation between the expression of PON1 and MSH2 was the strongest (R = 0.37, *P* = 4E-13).

### Constructing nomogram for prognostic evaluation of TACE treated patients

**Fig. 7:**
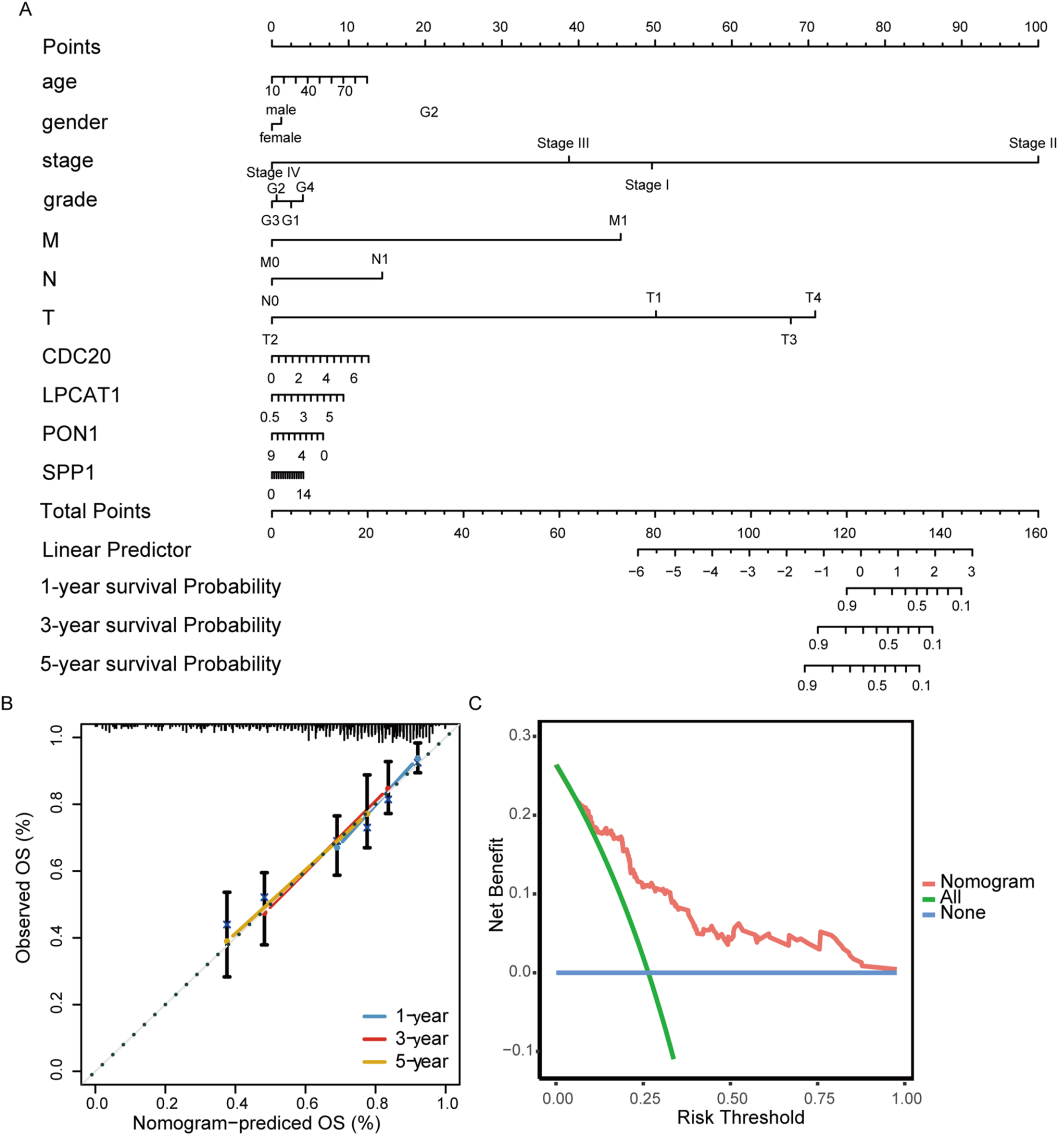
Construction of nomogram based on key genes and clinical characteristics. **A** Nomogram for predicting the overall survival at 1, 3, and 5 years. **B** Calibration curve for consistency between 1-year, 3-year, and 5-year nomogram-predicted survival and actual survival. **C** Decision curve analysis evaluated the clinical benefit of nomogram model

A nomogram based on key genes and clinical characteristics was constructed using the TCGA-LIHC dataset to evaluate the 1-year, 3-year, and 5-year survival probabilities of HCC patients. By scoring the above characteristics, patients with a higher total score had a worse 1-year, 3-year, and 5-year survival probability (Fig. 7A). Calibration curves indicated that the nomogram model has a good prognostic ability (Fig. 7B). DCA showed that the nomogram model was more effective in clinical utility for patients (Fig. 7C).

### Construction of TF regulatory network

**Fig. 8:**
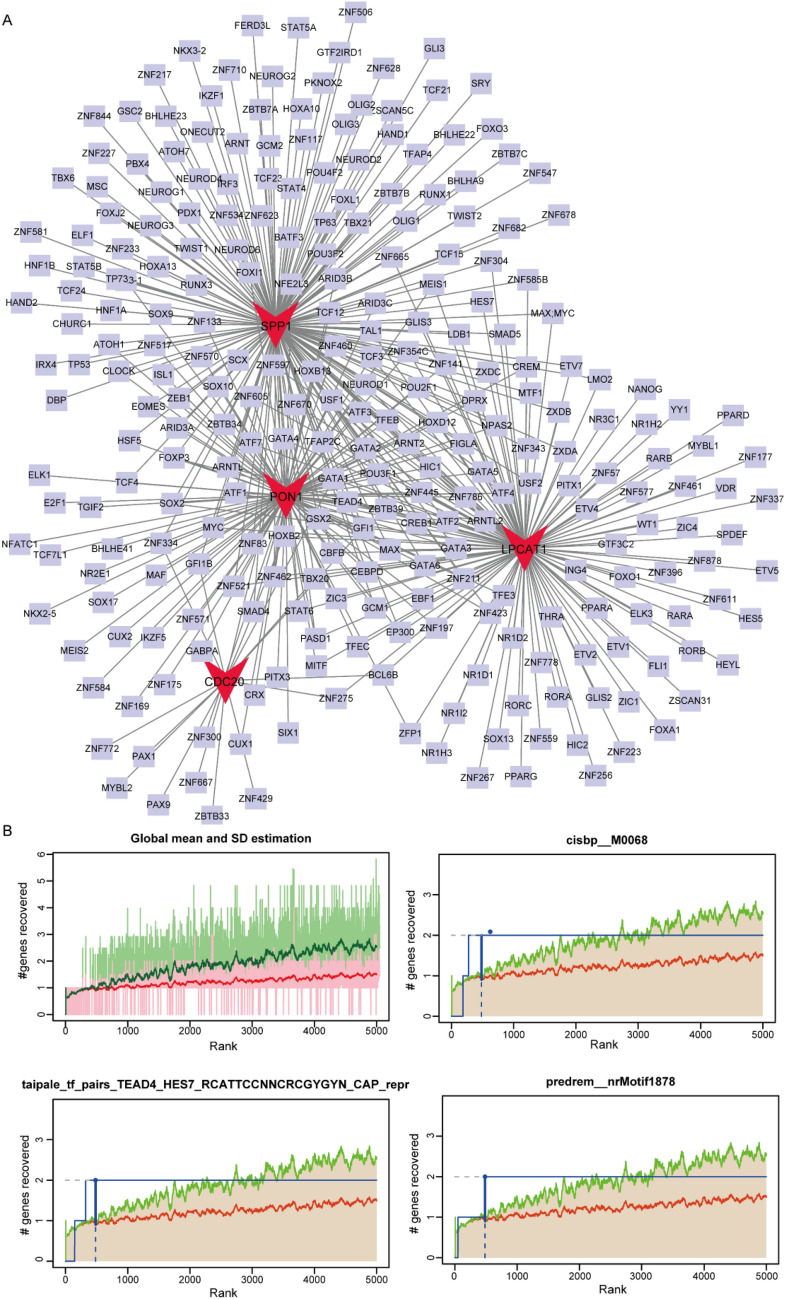
Transcription factor regulatory network. **A** The TF regulatory network. The red V-shape represents key genes, and the purple square represents TFs. **B** The recovery curve graph shows the results of motif enrichment analysis. The horizontal axis represents the ranking of motifs, while the vertical axis represents the recovery rate

Since TFs affect cell function by regulating gene expression [27], the relationship between key genes and TFs was analyzed. It was found that CDC20, LPCAT1, PON1 and SPP1 were regulated by multiple TFs (Fig. 8A). Motif enrichment analysis of these TFs showed that the motif with the highest NES was annotated as cisbp M0068 (NES: 8.69), followed by “taipale _ tf _ pairs _ TEAD4 _ HES7 _ RCATTCCNNCRCGYYN _ CAP _ repr” (NES: 8.62), and “predrem'nrMotif1878” (NES: 8.34) (Fig. 8B). The genes enriched in “taipale _ tf _ pairs _ TEAD4 _ HES7 _ RCATTCCNNCRCGYYN _ CAP _ repr” were LPCAT1 and SPP1, and the predicted upstream TFs were HES7 and TEAD4.

### Construction of ceRNA regulatory network

In total, 25 HCC-related miRNAs were obtained from the HMDD database. Then, 23 miRNAs were predicted to interact with 4 key genes in miRWalk database. Subsequently, 25 HCC-related miRNAs and 23 miRNAs in miRWalk database were overlapped to obtain 2 miRNAs and 2 miRNA-mRNA interaction pairs (Figure S3A). In DIANA-LncBase Predicted v.2 database, 68 lncRNAs were predicted to interact with these two miRNAs, and 74 lncRNA-miRNA interaction pairs were obtained. By combining the lncRNA-miRNA pairs and miRNA-mRNA pairs, the complex ceRNA regulatory network was visualized using cytoscape software (Figure S3B).

### Drug sensitivity analysis

**Fig. 9:**
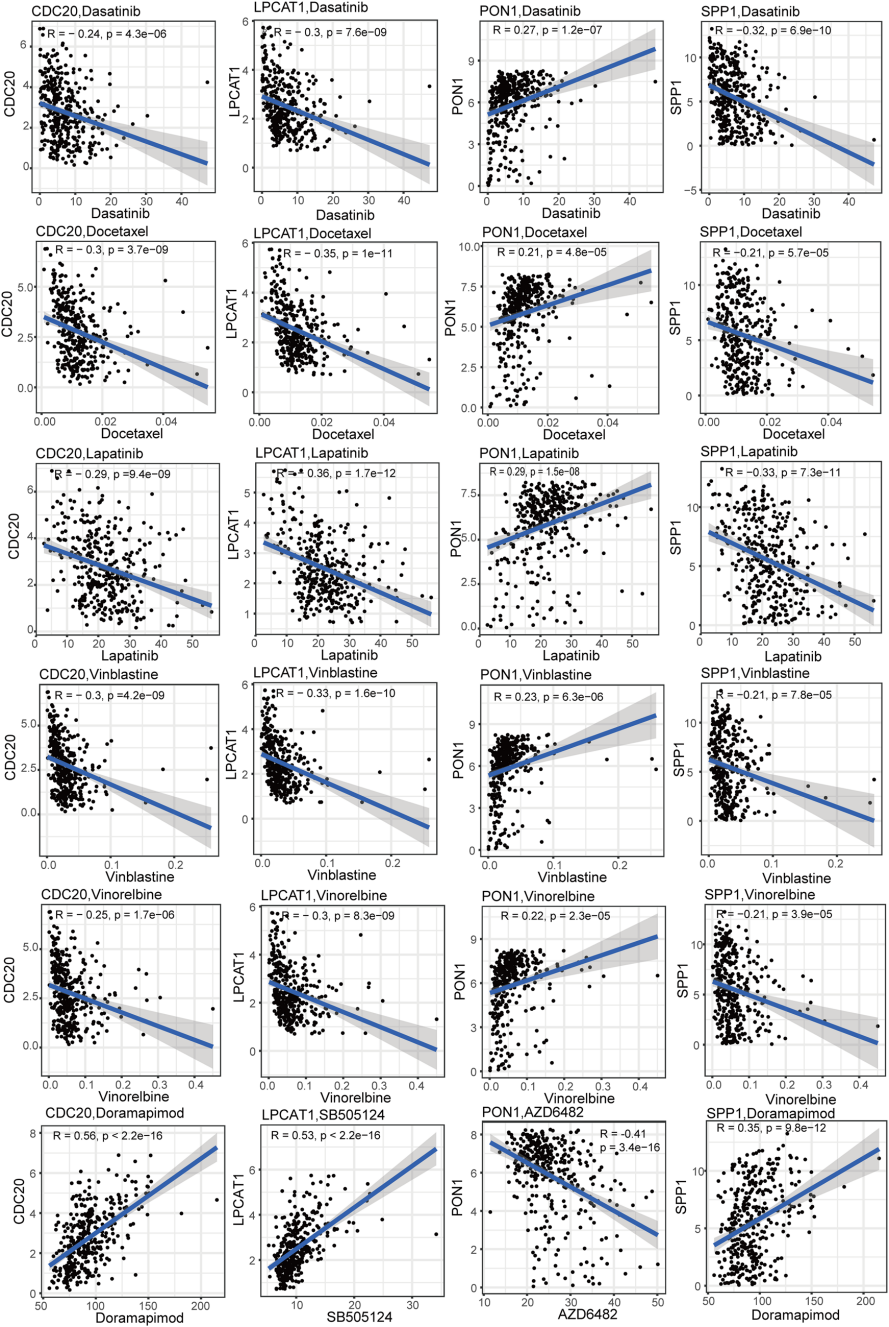
Drug sensitivity analysis of the correlation between chemotherapy drugs and 4 key genes expression levels based on the GDSC database

Further drug sensitivity analysis showed that the IC_50_ levels of 5 commonly used chemotherapy drugs (Dasatinib, Docetaxel, Lapatinib, Vinblastine, Vinorelbine) were significantly correlated with the expression levels of 4 key genes (Fig. 9). Specifically, they were negatively correlated with the expression of CDC20, LPCAT1, and SPP1, and positively correlated with the expression of PON1. In addition, the IC_50_ of Doramapimod was strongly positively correlated with the expression of CDC20 (R = 0.56, *P* < 2.2E-16) and SPP1 (R = 0.35, *P* = 9.8E-12), the IC_50_ of SB505124 was strongly positively correlated with the expression of LPCAT1 (R = 0.53, *P* < 2.2E-16), and the IC_50_ of AZD6482 was strongly negatively correlated with the expression of PON1 (R = -0.41, *P* = 3.4E-16) (Fig. 9).

### Verification of the expression level of four key genes

**Fig. 10:**
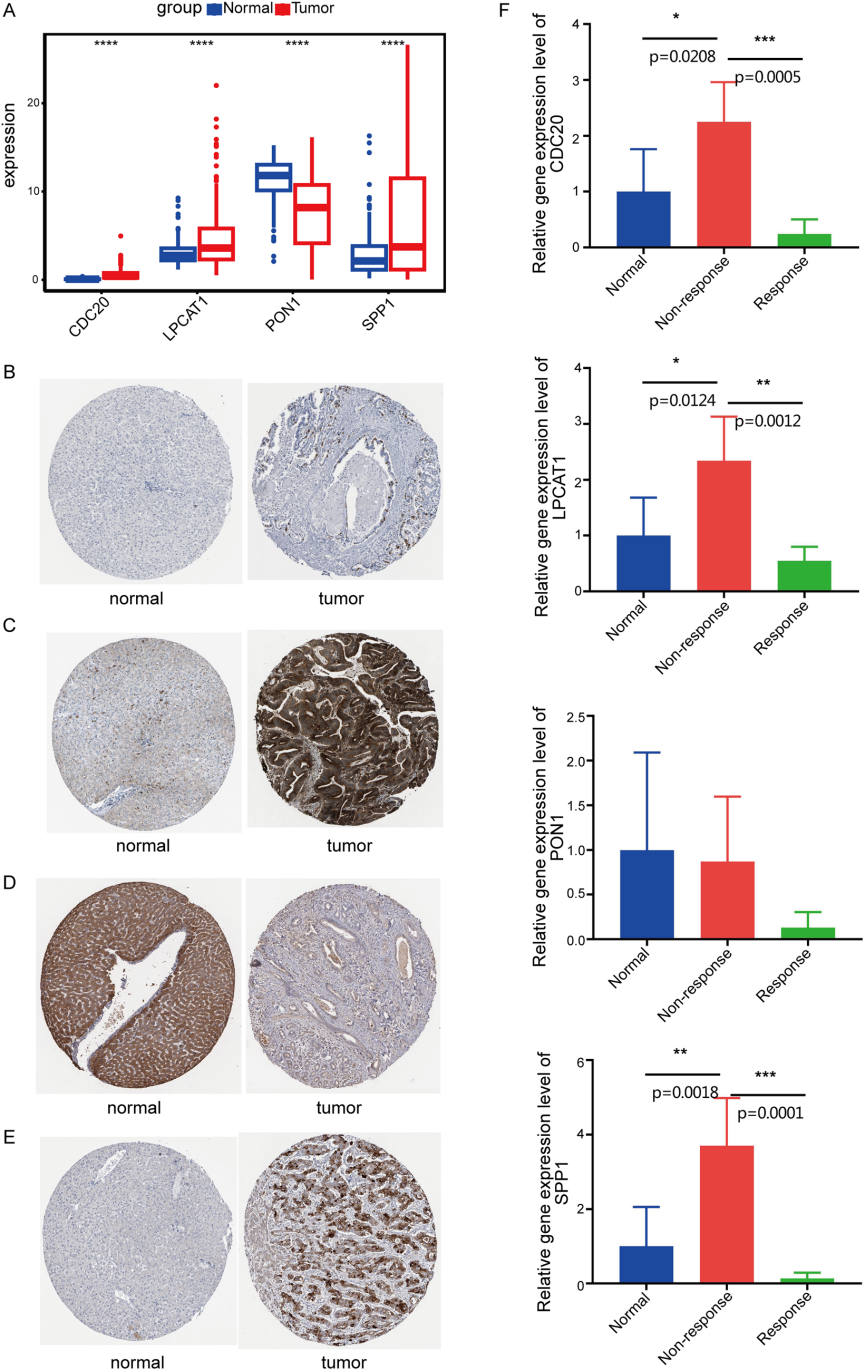
Verification of four key genes, CDC20, LPCAT1, PON1, SPP1. **A** Box plot shows the validation of four key genes expression between tumor and normal tissues based on the GSE25097 dataset. **B**-**E** The protein expression levels of CDC20 (**A**), LPCAT1 (**B**), PON1 (**C**), and SPP1 (**D**) between normal and tumor tissues from the HPA database. **F** RT-qPCR results of CDC20, LPCAT1, PON1, and SPP1

In GSE25097 dataset, compared with normal tissues, the expressions of CDC20, LPCAT1, and SPP1 were significantly upregulated, while PON1 was significantly downregulated in tumor tissues, which was consistent with the results in TCGA (Fig. 10A). The IHC results based on the HPA database were consistent with the above expression trends (Fig. 10B-E). In addition, RT-qPCR results showed that the mRNA levels of CDC20, LPCAT1, and SPP1 were higher in the TACE non-response group than in the normal and TACE response groups, while there was no significant difference in PON1 between the TACE non-response and normal/TACE response groups (Fig. 10F).

## Discussion

TACE has been widely recognized as a primary treatment for advanced-stage HCC [28, 29]. However, the sensitivity to TACE treatment varies from patient to patient [11, 30]. In recent years, metabolic reprogramming, as one of the important characteristics of tumors, has provided a new perspective for studying the sensitivity of TACE treatment in HCC [11, 12]. Our study analyzed the metabolic reprogramming-related genes in HCC after TACE treatment, and identified four key genes (CDC20, LPCAT1, PON1, and SPP1). It was found that their expression levels were closely related to tumor immune, HCC-related genes, the disease progression and survival prognosis of patients, and chemotherapy drug sensitivity. In addition, TF regulatory network and ceRNA regulatory network were constructed. Finally, their expression was validated by GSE25097 and HPA databases and in *vitro* RT-qPCR.

Targeting abnormal metabolic pathways can enhance the sensitivity of HCC to existing treatment methods, such as TACE [12, 31]. Our study identified 4 key genes, CDC20, LPCAT1, PON1, and SPP1, that were associated with metabolic reprogramming and TACE treatment sensitivity. CDC20 is a cell cycle protein that belongs to the activation protein of substrates in APC/C complexes [32]. It was reported that high expression of CDC20 regulated epithelial-mesenchymal transition (EMT) to promote HCC progression [33]. A study on reversing HCC drug resistance showed that APC/C-CDC20 complex-mediated degradation of p21 ubiquitination was prevented by CMTM6, affecting HCC response to TACE treatment [34]. Zhao et al. found that high expression of CDC20 may be associated with enhanced radiotherapy resistance in HCC [35]. LPCAT1 is a key enzyme involved in lipid metabolism remodeling, and its elevated levels are associated with poor prognosis and disease progression in HCC patients [36]. It was reported that highly expressed LPCAT1 promoted EMT in HCC by activating the Wnt/β-catenin signaling pathway [37]. At present, studies on LPCAT1 and tumor resistance have been reported in lung adenocarcinoma and breast cancer [38, 39]. However, the role of CDC20 and LPCAT1 in TACE treatment sensitivity of HCC had not been studied.

PON1 is an antioxidant enzyme [40]. A study revealed that downregulation of PON1 was an indicator for predicting poor survival prognosis and high recurrence rate in HCC patients [41]. It was reported that PON1 was associated with oxidative stress status and resistance to neoadjuvant chemoradiotherapy in patients with rectal cancer [42]. SPP1 is an integrin binding phosphorylated glycoprotein [43], which may be involved in the metabolic pathway of HCC [44]. It was reported that SPP1-mediated anoikis resistance and immune escape promoted invasion and metastasis of HCC [45]. To our knowledge, there have been no relevant reports on the PON1 and SPP1 in TACE treatment of HCC. Our study found that compared with the TACE treatment response group, the expression of CDC20, LPCAT1, and SPP1 was upregulated, and PON1 was downregulated in the TACE treatment non-response group. Moreover, compared with normal tissues, tumor tissues also exhibited this trend. The same was true for advanced-stage patients and patients with poor prognosis. In addition, the nomogram for predicting the prognosis of TACE treatment was constructed based on four key genes' expression and patients' clinical characteristics. Calibration curves revealed that nomogram-predicted overall survival was highly predictive in HCC patients. This indicated that the four key genes were closely related to the occurrence, progression, survival prognosis, and TACE treatment sensitivity of HCC.

The tumor immune microenvironment plays an important role in influencing the sensitivity of tumor treatment. It is of great significance to understand the roles and functions of various immune cells, as well as their relationship with tumor drug resistance [46]. Liang et al. systematically reviewed that immune microenvironment regulation (abnormal immune cell infiltration /suppressive factor expression) due to hypoxic stress was closely related to HCC progression and prognosis [47]. Zhou et al. found that tumor associated neutrophils promoted tumor progression and sorafenib resistance by releasing cytokines CCL2 and CCL17, recruiting macrophages and Tregs into HCC [48]. Peng et al. revealed that monocytes can regulate the entry of neutrophils into the HCC microenvironment and inhibit their apoptosis by activating glycolysis to mediate CXCL2 and CXCL8 production [49]. This interaction may affect the immune status of the tumor microenvironment, leading to the development of tumor drug resistance. NK cell dysfunction led to resistance to PD-1 immunotherapy in HCC patients [50]. Interestingly, our study found that the expression levels of CDC20, LPCAT1, SPP1, and PON1 were significantly correlated with the infiltration levels of various immune cells, including "Macrosphages M0", "T cell regulators (Tregs)", "Monocytes", and "NK cell resting". Among them, "T cell regulators (Tregs)" can promote immune escape [51], "Monocytes" can activate glycolysis [49]. This suggested that high expression of CDC20, LPCAT1, and SPP1, as well as low expression of PON1, may affect the immune escape and cell metabolism of tumor cells, reducing TACE treatment sensitivity.

Understanding the abnormal expression of tumor-related genes is crucial to predict and improve tumor treatment sensitivity [52]. Our study conducted a correlation analysis between HCC-related genes and four key genes. It was found that the expression of CDC20 had the strongest positive correlation with BRCA1 expression, and the expression of PON1 had the strongest negative correlation with MSH2 expression. BRCA1 was a tumor suppressor gene, and its encoded product was involved in DNA damage homologous recombination repair [53]. BRCA1 participated in tumor immune suppression and T lymphocyte infiltration of HCC [54]. Cho et al. found that CDC20 regulated the stability and ubiquitination of RAP80 in the BRCA1 complex, affecting the recruitment of BRCA1 at DNA damage sites and the DNA damage response in HeLa cells [55]. MSH2 was a key participant in the human DNA mismatch repair system [56], and its dysregulation was associated with HCC development [57]. However, the interaction between PON1 and MSH2 has not been reported. This finding suggests that the role of CDC20 and PON1 in TACE treatment sensitivity of HCC may be related to DNA repair damage-related genes BRCA1 and MSH.

TF regulatory network revealed that 4 key genes were regulated by multiple TFs. Motif enrichment analysis predicted that the upstream TFs of LPCAT1 and SPP1 were HES7 and TEAD4. HES7 is one of the downstream target genes of Notch signaling pathway. A previous study had suggested that HES7 transcriptional inhibition of Gli1 expression and Hedgehog signaling may cause drug resistance to PD-1 therapy in classical Hodgkin lymphoma [58]. TEAD4 is a transcriptional coactivator in the Hippo signaling pathway. It is reported that TEAD4 upregulated CDC25B in gastric adenocarcinoma, driving cell aggressiveness and inhibiting sensitivity to cisplatin therapy through cell adhesion [59]. However, there have been no reports on the study of HES7 and TEAD4 in HCC. Our study suggested that HES7 and TEAD4 may affect TACE treatment sensitivity in HCC by regulating LPCAT1 and SPP1 expression, and their interaction needs further verification. Furthermore, perturbations of ceRNA interactions may trigger or promote the development and progression of HCC [60]. Our study found 74 "lncRNA-miRNA" relationship pairs, which may affect the TACE treatment sensitivity in HCC by regulating the expression of PON1 and LPCAT1.

TACE is widely used as an interventional treatment for advanced HCC [4]. However, due to the treatment responsiveness varies from patient to patient, it is necessary to explore new additional treatment methods. Our study screened potential drugs based on the expression levels of four key genes and the IC_50_ values of drugs using the GDSC database. Correspondingly, five chemotherapy drugs were identified as candidates for TACE non-responders. Previous studies have shown that Dasatinib, Docetaxel, Lapatinib, Vinblastine, and Vinorelbine have anti-tumor activity in HCC [61–65]. This suggests that five chemotherapy drugs may have promising applications in TACE treatment non-responsive patients. Our study has laid a theoretical foundation for exploring the efficacy of chemotherapy combined with TACE treatment in the future.

Overall, the four key genes, CDC20, LPCAT1, PON1, and SPP1, may have great potential value in HCC clinical practice: (1) predict the response of HCC patients to TACE treatment by detecting gene expression in blood or tissue, identify optimal candidate patients in advance to improve treatment success; (2) serve as prognostic assessment and monitoring tools for TACE treatment, enabling timely evaluation of treatment efficacy and adjustment of treatment plans through regular monitoring their level changes; (3) provide reference for personalized medication for HCC patients and assist doctors in selecting appropriate chemotherapeutic drugs. However, there are some shortcomings in this study. The RT-qPCR validation was limited by a small sample size, which may introduce sampling bias and reduce result reliability. Future studies need to expand the sample size to further confirm these findings. Although our study identified key genes for TACE treatment sensitivity in HCC via gene expression databases, other biomarkers like functional hemodynamic parameters and structural lymphatic vessel indicators were equally crucial for predicting patient outcomes and prognosis [66–68]. Future studies should explore integrating these biomarkers to optimize TACE precision therapy in HCC. Moreover, we will further carry out corresponding verification work on cell and animal models to confirm the credibility and validity of this study.

## Conclusion

In this study, we investigated the differences in gene expression between normal tissues and tumor tissues, as well as TACE response and non-response groups, and intersected them with MRGs, successfully identifying four key genes (CDC20, LPCAT1, PON1, and SPP1). It was found that their expression levels were closely related to tumor immune, HCC-related genes, the disease progression and survival prognosis of patients, as well as chemotherapy drug sensitivity. In short, this study provides new biomarkers and a theoretical foundation for TACE treatment sensitivity in HCC. It provided a new perspective for developing individualized treatment plans for HCC patients.

## Supplementary Information

Supplementary material 1 (TIF 59631 KB)

Supplementary material 2 (TIF 87858 KB)

Supplementary material 3 (TIF 4512 KB)

Supplementary material 4 (DOCX 4033 KB)

Supplementary material 5 (DOCX 23 KB)

Supplementary material 6 (DOCX 17 KB)

## Data Availability

All data used and/or analyzed in this study are available from the corresponding author upon reasonable request.
