# Supplementary figures and images for: Identification of metabolic reprogramming-related key genes in hepatocellular carcinoma after transcatheter arterial chemoembolization treatment
Source: Discov Oncol. 2025 May 22;16:861. doi: 10.1007/s12672-025-02606-z (PMC12098233; doi:10.1007/s12672-025-02606-z)

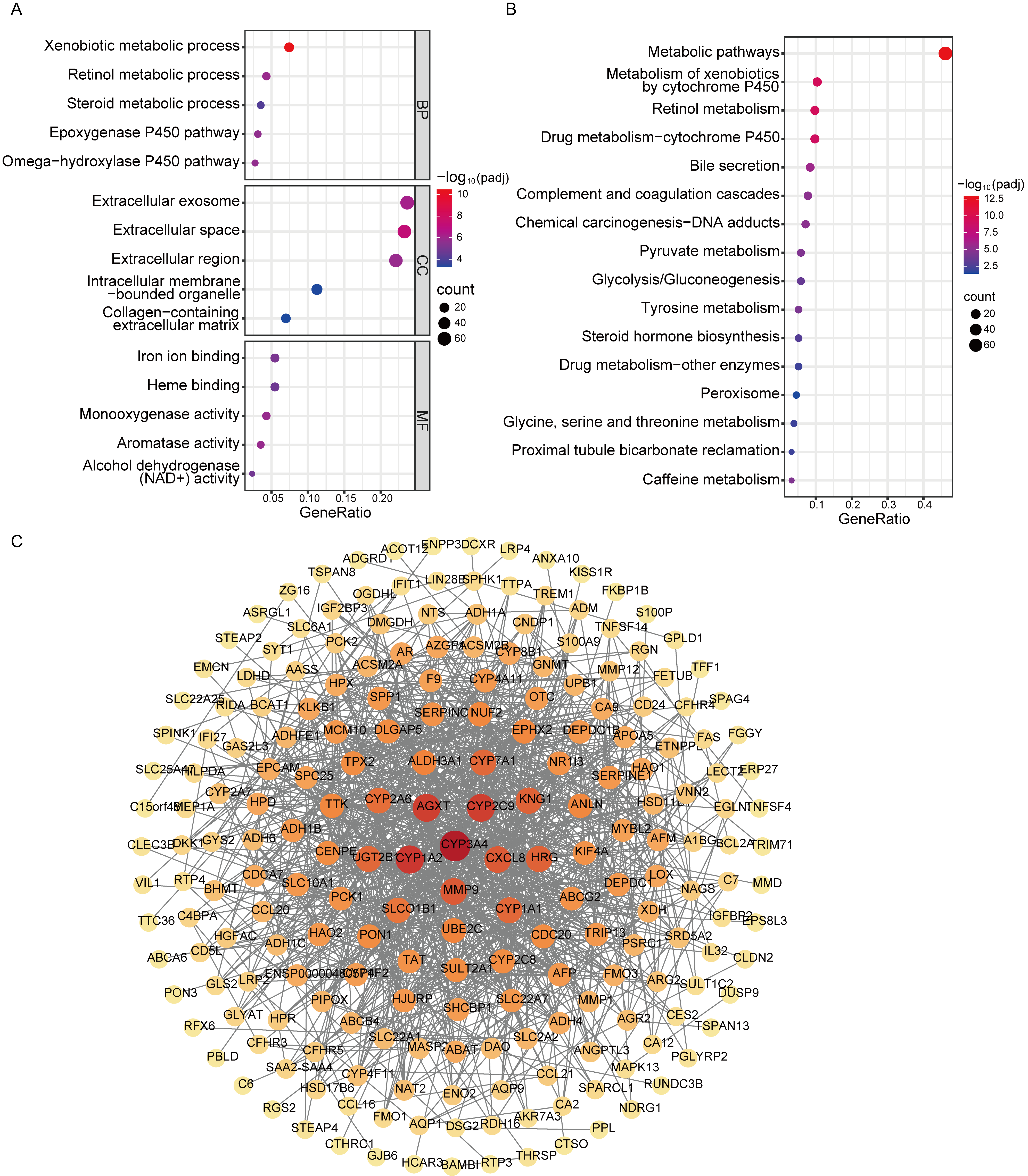

Supplement: Supplementary file 1 — Supplementary material 1 (TIF 59631 KB) [file 12672_2025_2606_MOESM1_ESM.tif]

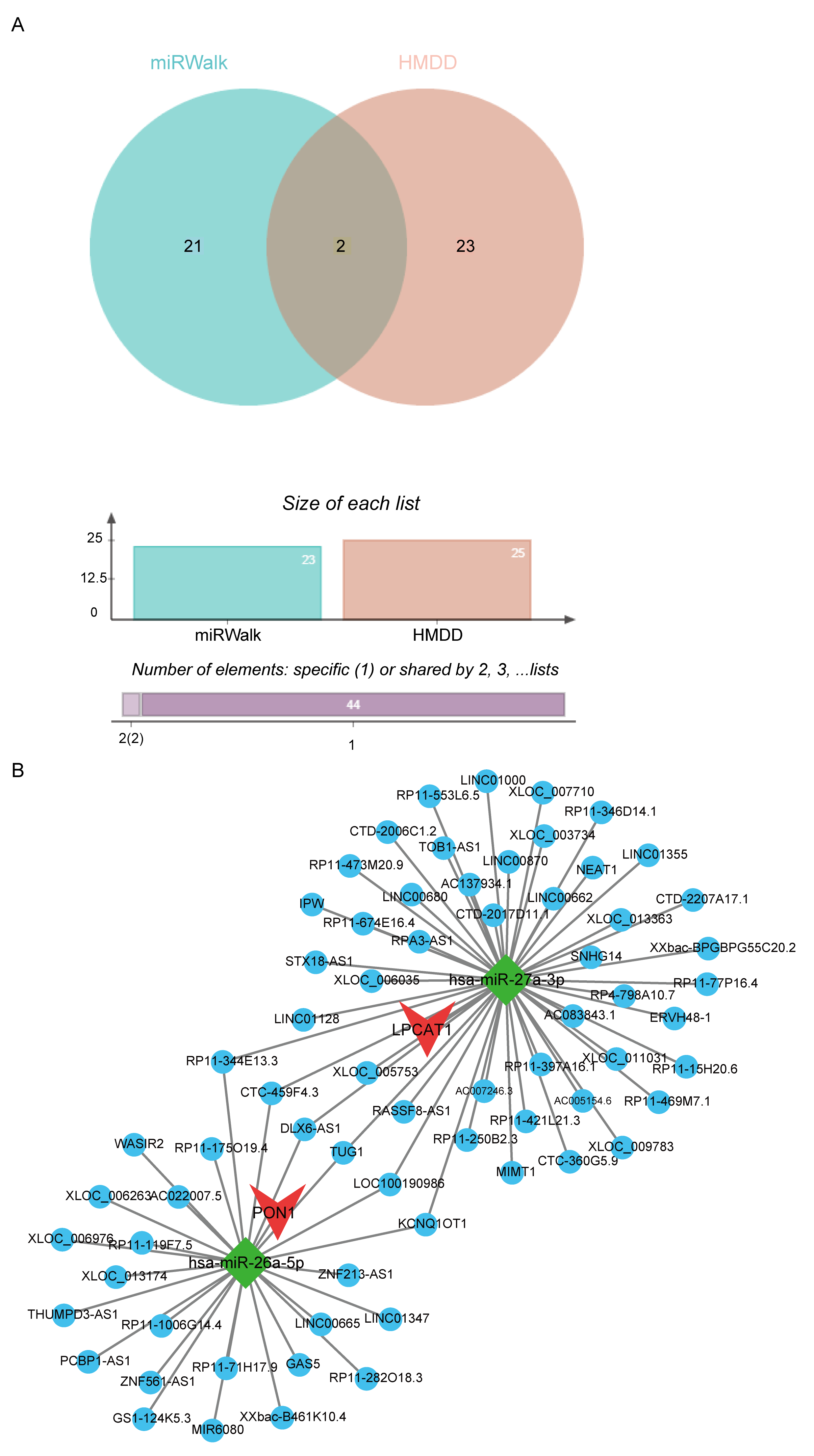

Supplement: Supplementary file 3 — Supplementary material 3 (TIF 4512 KB) [file 12672_2025_2606_MOESM3_ESM.tif]
