# Supplementary material for: Identification of metabolic reprogramming-related key genes in hepatocellular carcinoma after transcatheter arterial chemoembolization treatment
Source: Discov Oncol. 2025 May 22;16:861. doi: 10.1007/s12672-025-02606-z (PMC12098233; doi:10.1007/s12672-025-02606-z)
Supplement: Supplementary file 4 — Supplementary material 4 (DOCX 4033 KB) [file 12672_2025_2606_MOESM4_ESM.docx]

**Supplementary materials**

**
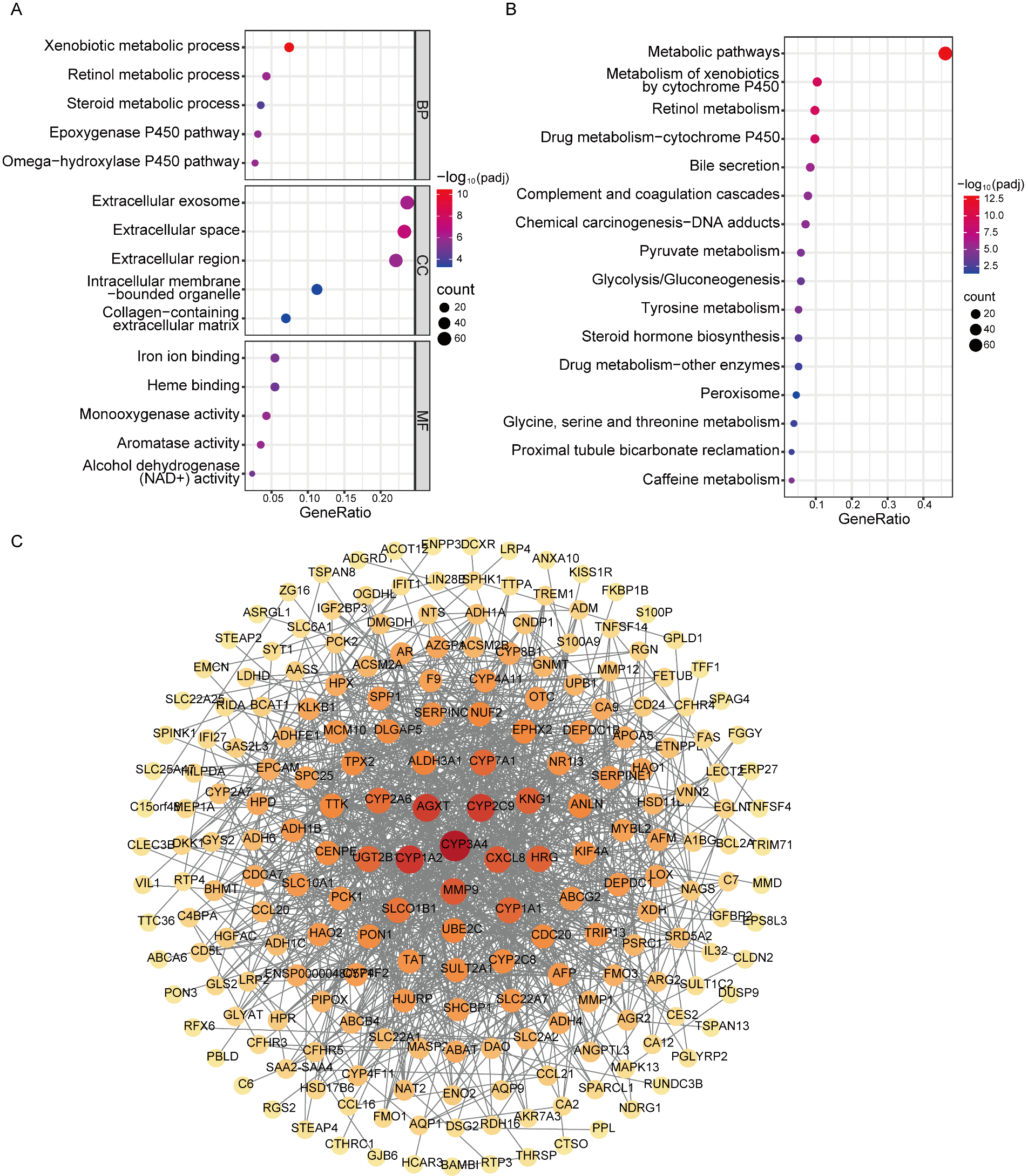
**

**Figure S1**. GO, KEGG pathways and PPI network analysis for DEGs.

(A) Bubble chart displays the top five BP, CC, MF. The X-axis represents the gene ratio, and the Y-axis represents the description of GO terms. The bubble size represents the number of genes enriched in the term, and the bubble color indicates -lg(padj), where padj is the adjusted p-value. BP, biological process; CC, cellular component; MF, molecular function. (B) Bubble plot shows KEGG pathways of DEGs. The bubble size represents the number of genes enriched in the pathway, while the bubble color indicates -lg(padj). (C) PPI network of DEGs. Nodes represent proteins, lines represent interactions between them, the size and color of nodes and lines are represented in descending order of degree value, from large to small and red to yellow.

**
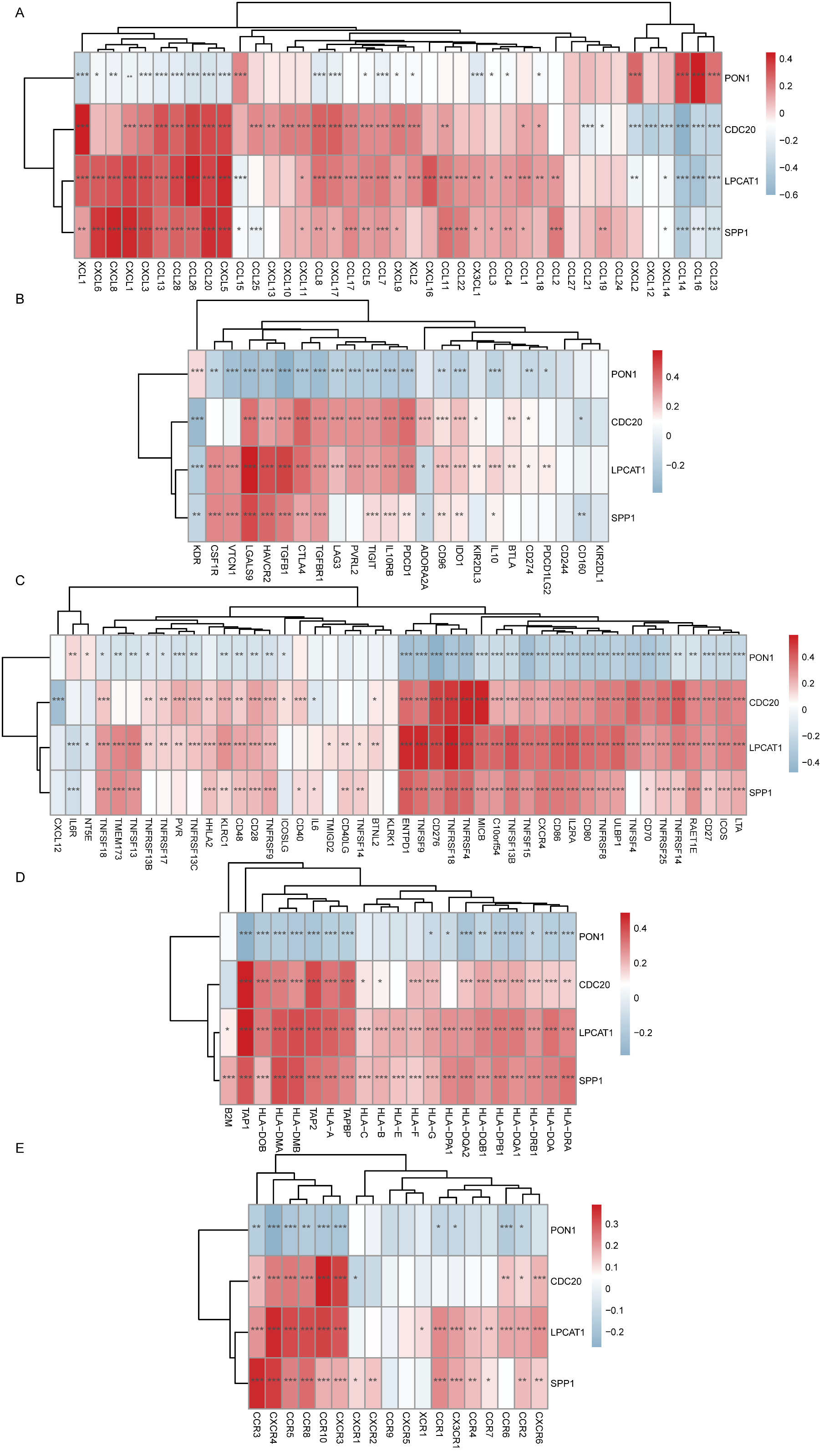
**

**Figure S2.** Correlation analysis between four key genes and immune-related genes.

(A-E) Heatmap shows the correlation between four key gene genes and chemokines (A), immunosuppressants (B), immune activators (C), MHC (D), and MHC receptors (E). Red to blue represents the change trend from positive correlation to negative correlation.


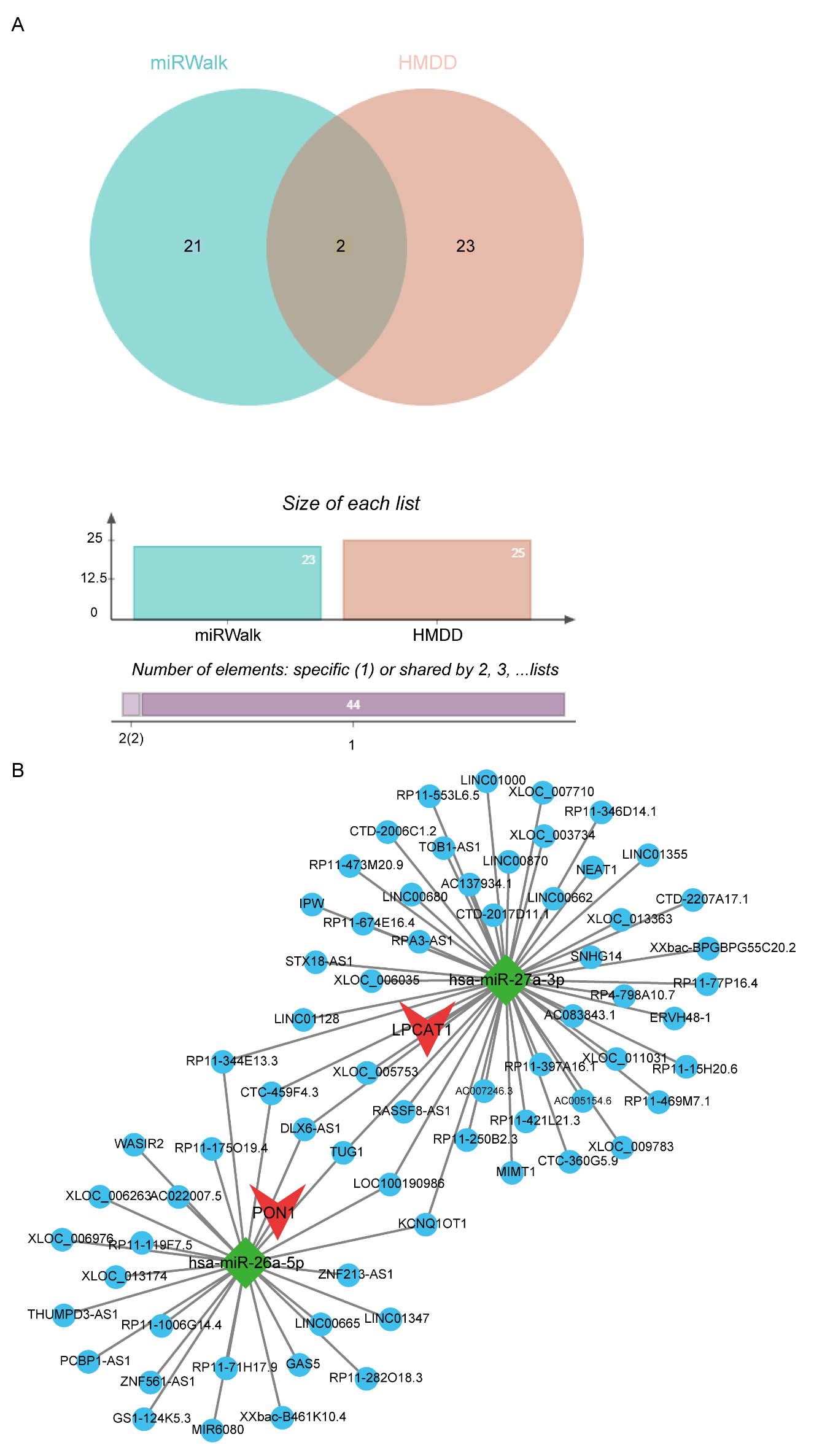


**Figure S3.** Construction of ceRNA regulatory network.

(A) The Venn diagram shows the intersection of 25 HCC-related miRNAs in the HMDD database and 23 miRNAs in the miRWalk database that interact with key genes. (B) The ceRNA regulatory network. The red V-shape represents mRNA, the green diamond represents miRNA, and the blue circle represents lncRNA.

**Table S1** Detailed clinicopathological characteristics of 15 subjects.

| Characteristics | Control  (n = 5) | TACE treatment (n = 10) | | P value |
| --- | --- | --- | --- | --- |
|  |  | Non-response  (n = 4) | Response  (n = 6) |  |
| Age (Years) | 59.40±9.07 | 58.80±8.63 | | 0.902^a^ |
| Sex (Male, %) | 4 (80.0) | 9 (90.0) | | 1^a^ |
| Body Mass Index (kg/m²) | 24.18±1.13 | 23.99±3.39 | | 0.906^a^ |
| alpha-fetoprotein (μg/L) | - | 509.23±566.71 | 386.59±481.11 | 0.722^b^ |
| stage (%) |  |  |  | 0.644^b^ |
| I | - | 0 (0.0) | 1 (16.7) |  |
| II | - | 1 (25.0) | 2 (33.3) |  |
| III | - | 1 (25.0) | 2 (33.3) |  |
| IV | - | 2 (50.0) | 1 (16.7) |  |
| T stage (%) |  |  |  | 0.615^b^ |
| T1 | - | 0 (0.0) | 1 (16.7) |  |
| T2 | - | 3 (75.0) | 3 (50.0) |  |
| T3 | - | 1 (25.0) | 2 (33.3) |  |
| N stage (%) |  |  |  | 0.859^b^ |
| N0 | - | 1 (25.0) | 3 (50.0) |  |
| N1 | - | 3 (75.0) | 3 (50.0) |  |
| M stage (%) |  |  |  | 1^b^ |
| M0 | - | 3 (75.0) | 4 (66.7) |  |
| M1 | - | 1 (25.0) | 2 (33.3) |  |
| Grade (%) |  |  |  | 0.082^b^ |
| G1 | - | 0 (0.0) | 3 (50.0) |  |
| G2 | - | 2 (50.0) | 3 (50.0) |  |
| G3 | - | 2 (50.0) | 0 (0.0) |  |
| Barcelona Clinic Liver Cancer stage |  |  |  | 0.859^b^ |
| B | - | 3 (75.0) | 3 (50.0) |  |
| C | - | 1 (25.0) | 3 (50.0) |  |
| Protein Induced by Vitamin K Absence (mAU/mL) | - | 121956.08±146286.13 | 7205.95±16686.18 | 0.085^b^ |
| Prothrombin time (s) | - | 12.78±1.47 | 12.62±2.01 | 0.897^b^ |
| Total bilirubin (umol/L) | - | 25.08±14.97 | 23.57±13.06 | 0.87^b^ |
| Albumin (g/L) | - | 35.52±2.62 | 38.90±5.23 | 0.272^b^ |
| Alanine transaminase (U/L) | - | 49.00±42.21 | 36.67±19.56 | 0.544^b^ |
| Aspartate aminotransferase (U/L) | - | 90.75±52.62 | 102.17±117.14 | 0.861^b^ |
| Creatinine (mg/dL) | - | 53.00±6.29 | 55.50±11.61 | 0.707^b^ |
| Tumor number |  |  |  | 0.615^b^ |
| 1 | - | 1 (25.0) | 2 (33.3) |  |
| 2 | - | 0 (0.0) | 1 (16.7) |  |
| 3 | - | 3 (75.0) | 3 (50.0) |  |
| Smoking (Yes, %) | - | 1 (25.0) | 2 (33.3) | 1^b^ |
| Hepatitis B virus infection (Yes, %) | - | 3 (75.0) | 5 (83.3) | 1^b^ |

^a^Control *vs*. TACE treatment; ^b^Non-response *vs*. Response. Unless otherwise specified, data are presented as mean ± SD.

**Table S2.** The sequence of primers in this study.

| **Gene** | **Species** | **Forward (5’−3’)** | **Reverse (5’−3’)** |
| --- | --- | --- | --- |
| GAPDH | Human | GGAGCGAGATCCCTCCAAAAT | GGCTGTTGTCATACTTCTCATGG |
| ACTB | Human | CATGTACGTTGCTATCCAGGC | CTCCTTAATGTCACGCACGAT |
| CDC20 | Human | GACCACTCCTAGCAAACCTGG | GGGCGTCTGGCTGTTTTCA |
| LPCAT1 | Human | ACATCCCGATCTGGGGAACT | GGCCACTTTCCGTTGGACT |
| PON1 | Human | GATACTGCCTAATGGACTGGC | GTGATCCCCAATTCCAACACT |
| SPP1 | Human | GAAGTTTCGCAGACCTGACAT | GTATGCACCATTCAACTCCTCG |
