# Supplementary material for: Identification of metabolic reprogramming-related key genes in hepatocellular carcinoma after transcatheter arterial chemoembolization treatment
Source: Discov Oncol. 2025 May 22;16:861. doi: 10.1007/s12672-025-02606-z (PMC12098233; doi:10.1007/s12672-025-02606-z)
Supplement: Supplementary file 5 — Supplementary material 5 (DOCX 23 KB) [file 12672_2025_2606_MOESM5_ESM.docx]

Table S1 Detailed clinicopathological characteristics of 15 subjects.

| Characteristics | Control  (n = 5) | TACE treatment (n = 10) | | P value |
| --- | --- | --- | --- | --- |
|  |  | Non-response  (n = 4) | Response  (n = 6) |  |
| Age (Years) | 59.40±9.07 | 58.80±8.63 | | 0.902^a^ |
| Sex (Male, %) | 4 (80.0) | 9 (90.0) | | 1^a^ |
| Body Mass Index (kg/m²) | 24.18±1.13 | 23.99±3.39 | | 0.906^a^ |
| alpha-fetoprotein (μg/L) | - | 509.23±566.71 | 386.59±481.11 | 0.722^b^ |
| stage (%) |  |  |  | 0.644^b^ |
| I | - | 0 (0.0) | 1 (16.7) |  |
| II | - | 1 (25.0) | 2 (33.3) |  |
| III | - | 1 (25.0) | 2 (33.3) |  |
| IV | - | 2 (50.0) | 1 (16.7) |  |
| T stage (%) |  |  |  | 0.615^b^ |
| T1 | - | 0 (0.0) | 1 (16.7) |  |
| T2 | - | 3 (75.0) | 3 (50.0) |  |
| T3 | - | 1 (25.0) | 2 (33.3) |  |
| N stage (%) |  |  |  | 0.859^b^ |
| N0 | - | 1 (25.0) | 3 (50.0) |  |
| N1 | - | 3 (75.0) | 3 (50.0) |  |
| M stage (%) |  |  |  | 1^b^ |
| M0 | - | 3 (75.0) | 4 (66.7) |  |
| M1 | - | 1 (25.0) | 2 (33.3) |  |
| Grade (%) |  |  |  | 0.082^b^ |
| G1 | - | 0 (0.0) | 3 (50.0) |  |
| G2 | - | 2 (50.0) | 3 (50.0) |  |
| G3 | - | 2 (50.0) | 0 (0.0) |  |
| Barcelona Clinic Liver Cancer stage |  |  |  | 0.859^b^ |
| B | - | 3 (75.0) | 3 (50.0) |  |
| C | - | 1 (25.0) | 3 (50.0) |  |
| Protein Induced by Vitamin K Absence (mAU/mL) | - | 121956.08±146286.13 | 7205.95±16686.18 | 0.085^b^ |
| Prothrombin time (s) | - | 12.78±1.47 | 12.62±2.01 | 0.897^b^ |
| Total bilirubin (umol/L) | - | 25.08±14.97 | 23.57±13.06 | 0.87^b^ |
| Albumin (g/L) | - | 35.52±2.62 | 38.90±5.23 | 0.272^b^ |
| Alanine transaminase (U/L) | - | 49.00±42.21 | 36.67±19.56 | 0.544^b^ |
| Aspartate aminotransferase (U/L) | - | 90.75±52.62 | 102.17±117.14 | 0.861^b^ |
| Creatinine (mg/dL) | - | 53.00±6.29 | 55.50±11.61 | 0.707^b^ |
| Tumor number |  |  |  | 0.615^b^ |
| 1 | - | 1 (25.0) | 2 (33.3) |  |
| 2 | - | 0 (0.0) | 1 (16.7) |  |
| 3 | - | 3 (75.0) | 3 (50.0) |  |
| Smoking (Yes, %) | - | 1 (25.0) | 2 (33.3) | 1^b^ |
| Hepatitis B virus infection (Yes, %) | - | 3 (75.0) | 5 (83.3) | 1^b^ |

^a^Control *vs*. TACE treatment; ^b^Non-response *vs*. Response. Unless otherwise specified, data are presented as mean ± SD.
