# Supplementary material for: Identification of metabolic reprogramming-related key genes in hepatocellular carcinoma after transcatheter arterial chemoembolization treatment
Source: Discov Oncol. 2025 May 22;16:861. doi: 10.1007/s12672-025-02606-z (PMC12098233; doi:10.1007/s12672-025-02606-z)
Supplement: Supplementary file 6 — Supplementary material 6 (DOCX 17 KB) [file 12672_2025_2606_MOESM6_ESM.docx]

**Table S2.** The sequence of primers in this study.

| **Gene** | **Species** | **Forward (5’−3’)** | **Reverse (5’−3’)** |
| --- | --- | --- | --- |
| GAPDH | Human | GGAGCGAGATCCCTCCAAAAT | GGCTGTTGTCATACTTCTCATGG |
| ACTB | Human | CATGTACGTTGCTATCCAGGC | CTCCTTAATGTCACGCACGAT |
| CDC20 | Human | GACCACTCCTAGCAAACCTGG | GGGCGTCTGGCTGTTTTCA |
| LPCAT1 | Human | ACATCCCGATCTGGGGAACT | GGCCACTTTCCGTTGGACT |
| PON1 | Human | GATACTGCCTAATGGACTGGC | GTGATCCCCAATTCCAACACT |
| SPP1 | Human | GAAGTTTCGCAGACCTGACAT | GTATGCACCATTCAACTCCTCG |
